# Evaluating the reliability and readability of online information on osteoporosis

**DOI:** 10.20945/2359-3997000000311

**Published:** 2020-11-09

**Authors:** Ozan Volkan Yurdakul, Mehmet Serkan Kilicoglu, Fatih Bagcier

**Affiliations:** 1 Bezmialem Vakıf University Faculty of Medicine Department of Physical Medicine and Rehabilitation Fatih Istanbul Turkey Bezmialem Vakıf University, Faculty of Medicine, Department of Physical Medicine and Rehabilitation, Fatih, Istanbul, Turkey; 2 Biruni University Faculty of Medicine Department of Physical Medicine and Rehabilitation Zeytinburnu Istanbul Turkey Biruni University, Faculty of Medicine, Department of Physical Medicine and Rehabilitation, Zeytinburnu, Istanbul, Turkey

**Keywords:** Osteoporosis, Google, internet, web pages

## Abstract

**Objective:**

Internet usage for obtaining health-related information is widely popular among patients. However, there are still concerns about the reliability and comprehensibility of online information. The purpose of this study is to investigate the reliability and readability of osteoporosis-related websites.

**Materials and methods:**

On April 2, 2020, we searched the term “osteoporosis” on Google (https://www.google.com). We evaluated the first 200 uniform resource locators (URLs) in the query results regarding typology, the *Journal of the American Medical Association* (*JAMA*) scores, Health on the Net Foundation Code of conduct (HONcode) certification, Flesch–Kincaid Grade (FKG), and Simple Measure of Gobbledygook (SMOG) scores. The *JAMA* scoring system and HONcode stamp were used for assessing the reliability, whereas FKG and SMOG scores were used to assess the readability of online information.

**Results:**

Of the 151 analyzed websites, 57 (37.7%) were classified as highly reliable, and 19 (12.6%) were assigned with HONcode certification. The average FKG scores (8.81 ± 2.21) and SMOG scores (7.63 ± 1.81) were below the recommended grade, which is considered as easily readable. High reliable information was found to have higher readability scores, thereby representing the difficulty of readability. We observed a weak correlation between the increased reliability of information and decreased readability.

**Conclusion:**

Osteoporosis-related content on the internet generally has low reliability. High-reliable information is available online in scientific published materials, health portals, and news. Although the readability of the overall material is acceptable, the high-reliable websites still require high literacy and comprehension skills.

## INTRODUCTION

**O**steoporosis is a disease that is characterized by low bone mass, deterioration of bone tissue, and disruption of bone microarchitecture, further leading to compromised bone strength and an increased risk of fractures (1). Osteoporosis is the most common bone disease in humans, which represents a significant health problem. It usually remains silent until the occurrence of fractures, which may cause secondary health problems, including death (2). Aging, sex steroid deficiency, glucocorticoids, reduced bone quality, disruption of microarchitectural integrity are some risk factors associated with an increased risk of osteoporotic fractures (3). An adequate intake of serum calcium and vitamin D, decreasing consumption of alcohol and caffeine, regular exercise, prevention of falls, and pharmacologic therapy are used for the management of osteoporosis (4).

With the wide usage of the internet, there is easier accessibility of online information regarding diseases, drugs, treatment choices, and surgical procedures. It has been demonstrated that half of the adult population in the United States uses the internet to obtain health-related information (5). The main reasons for patients using online information are to verify information from doctors, find answers to their questions, and seek alternative treatment options (6). Modern-day patients are keen to search the internet after a specific diagnosis. The chronic course of osteoporosis and its treatment may incite patients to seek alternative sources of information, and clinicians may encounter patients who had obtained information from online sources. Although the availability of online sources may be useful for patients’ participation, there are increasing concerns about the quality and accuracy of online health-related information (7-9). While the online content varies from high-reliable to misleading information, there is no specific mechanism to assess the accuracy and reliability of online health-related sources. Patients with low health literacy skills may have difficulty in assessing the health-related information. Previous research has documented that many individuals are unaware of the risk factors of osteoporosis and potentially debilitating consequences (10,11). Hence, patients’ education is an essential component for the effective prevention and management of osteoporosis. Physicians need to refer the patients to accurate sources.

To our knowledge, there is only one study that has assessed the readability and reliability of online materials on osteoporosis and evaluated 27 websites in 2005 (9). Because the information on the internet is rapidly growing, an update of online information on osteoporosis with more websites is a necessity. In this manner, the purpose of this study is to evaluate the reliability and readability of websites on osteoporosis. Additionally, this study aimed to determine the website typologies that provide high-reliable information on osteoporosis.

## MATERIALS AND METHODS

### Study design and data collection

For this descriptive study, we searched websites on Google (https://www.google.com) by using the search term “osteoporosis” on April 2, 2020. This term was chosen to contain a larger website sample. Recently, Google is the leading search engine in January 2020 with a market share of 87.35% (12); therefore, Google was used as the main search engine for our study. We signed out from Google account and also deleted cookies and browser history before the search to avoid the effects of previous internet use (such as Google Ads) on search results. We recorded the first 200 uniform resource locators (URLs) of websites consistently similar to the previous studies (13–16). Internet users typically review websites on the first page of query results (17). The first page on Google includes ten websites; therefore, the first ten websites are considered as the most viewed.

The following exclusion criteria were employed for our study: 1) websites that required subscription or registration, 2) inaccessible websites, 3) websites that contained no information about osteoporosis, 4) repetitive websites, 5) websites that contain non-readable information such as videos or sound recordings, and 6) websites in languages other than English.

If no criteria appeared on the website's homepage while evaluating the websites, then we applied a three-click rule. The three-click rule is a rule of web design regarding the design of website navigation. This rule recommends that the website's user should be able to discover any information with no more than three clicks of a mouse. It is based on the belief that the website users will become frustrated and often leave if they cannot find the information within the three clicks (18). If there were many relevant topics on URL indexed in Google, per the three-click rule, then we analyzed the most current topic if the topics were sorted according to date or the first topic if no clue about the date was explicit on URL.

This study did not assess any human participants or animals. Therefore, the approval of the ethics committee was not required for this study.

### Types of websites

Websites were divided into the following eight categories: 1) commercial (websites that sell products or provide services to generate profits), 2) government (websites created, administered, or regulated by an official government agency), 3) health portals (websites that provide health-related information on various topics), 4) news (newspaper or magazine websites that are created to provide news and information), 5) nonprofit (charitable/supportive/educational websites that were not established to generate profits), 6) professional (websites created by organizations or individuals with professional medical qualifications), 7) scientific publishing (online scientific journals or academic publishing), and 8) others (websites that could not be classified in any type of typology). Two independent assessors (OVY, MSK) evaluated the types of websites. If there was an inconsistency regarding the evaluation of the website, then a third independent assessor (FB) evaluated the website and made the final decision.

### The *Journal of the American Medical Association* (*JAMA*) score

The *JAMA* scoring system comprised four parameters: 1) authorship (authors and contributors, their affiliations, and relevant credentials should be provided), 2) attribution (references and sources for all content should be listed clearly, and all relevant copyright information should be adequately noted), 3) disclosure (website's “ownership” should be prominently and fully disclosed, as should any sponsorship, advertising, underwriting, commercial funding arrangements or support, or potential conflicts of interest), and 4) currency (dates on which the content was posted and updated should be indicated) (7). The absence of each parameter is scored as 0 points, and its presence as 1 point. The maximum possible total score is 4, where the minimum score is 0. A website with ≥3 points is considered as high-reliable, whereas ≤2 is considered as low-reliable (19). Two independent investigators (OVY and MSK) evaluated *JAMA* scores. If the sought information about a parameter was written in any location on URL or was available with a simple click from the URL, then the score was considered as 1 for the parameter. If there was a discrepancy among *JAMA* scores, then a third independent assessor (FB) evaluated and finalized the website.

### Health on the Net Foundation Code of conduct (HONcode) certification

Health on the Net Foundation (a nonprofit organization) issued a code of conduct (HONcode) for medical and health websites to address the reliability and usefulness of medical information on the internet (20). The code only states that the site holds to the standards so that the readers can know the source and purpose of the presented medical information. The certification process evaluates eight main topics comprising authoritative, complementarity, privacy, attribution, justifiability, transparency, financial disclosure, and advertising policy (21). The HONcode is voluntary, which means that webmasters and information providers can apply for HONcode certification. However, HONcode certification is costly; therefore, its usability may be limited. We examined whether the HONcode stamp exists on the homepage or relevant URL.

### Readability

We used an online tool to assess the Flesch-Kincaid Grade (FKG) and the Simple Measure of Gobbledygook (SMOG) scores of websites (https://www.webfx.com/tools/read-able/). The “FKG Level Formula” presents a score as a US grade level, thereby making it easier for teachers, parents, librarians, and others to judge the readability level of various books and texts. The FKG evaluates the average sentence length and average syllables per word in the calculation of the readability score (22). The SMOG grade is a measure of readability that estimates the years of education needed to understand a piece of writing. In addition to parameters in the FKG scoring system, the SMOG grade evaluates the number of polysyllabic words in 30 sentences (23). The SMOG is widely used, particularly for checking health-related texts (24).

### Statistical analysis

We used IBM SPSS Statistics v21.0 statistical software (Armonk, NY, USA) to perform all statistical analyses. Moreover, we used the Shapiro-Wilk test to test the variable distribution. Descriptive statistics were indicated as “mean ± standard deviation” and “median, minimum − maximum” for quantitative variables, whereas “frequency and percentage [n (%)]” were indicated for categorical variables. We used Cohen's κ to determine the inter-rater reliability for categorical or ordinal items, and the Mann–Whitney *U* test to compare two groups with non-normally distributed variables. We employed the Spearman's rank correlation analysis to assess the correlations among non-normal distributing variables. The relations were interpreted as highly correlated when r was ≥ 0.60, moderately correlated when r was between 0.30 and 0.60, and weakly correlated when r was ≤ 0.30 (25). A p-value of less than 0.05 was considered statistically significant for this study.

## RESULTS

**Figure 1:**
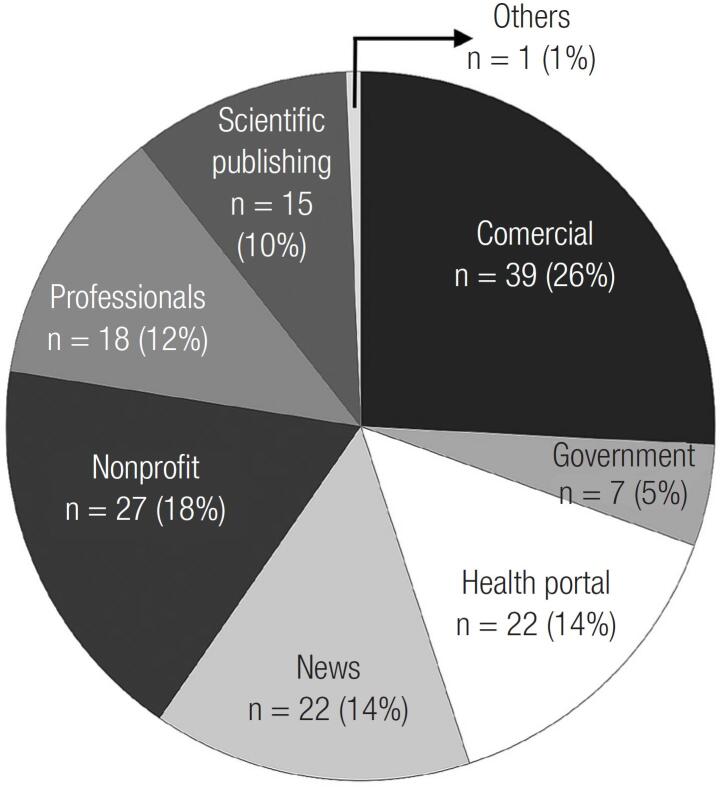
Types of websites in the whole search.

Among the 151 websites evaluated, based on typologies, the websites were classified as commercial (n = 39, 26%), nonprofit (n = 27, 18%), health portals (n = 22, 14%), news (n = 22, 14%), professionals (n=18, 12%), scientific publishing (n = 15, 10%), government (n = 7, 5%), and others (n = 1, 1%) (Figure 1).

The inter-rater reliability for determining the typology was almost excellent (Cohen's kappa coefficient (κ) = 0.954). It was demonstrated that internet users typically access and read websites on the first page of the search engine (17). Google indexes ten websites on its first page. When we compared the first ten pages and the remaining webpages in terms of typology, we observed no significant difference (p = 0.206). Table 1 presents the different typologies of websites searched in our study.

**Table 1:** Types of websites in the study

Type	Initial 10 n (%)	Websites other than initial 10 n (%)	p
Commercial	1 (10.0%)	38 (27.0%)	0.206*
Government	2 (20.0%)	5 (4.0%)	
Health portal	1 (10.0%)	21 (15.0%)	
News	3 (30.0%)	19 (13.0%)	
Nonprofit	2 (20.0%)	25 (18.0%)	
Professionals	1 (10.0%)	17 (12.0%)	
Scientific publishing	0 (0.0%)	15 (11.0%)	
Others	0 (0.0%)	1 (1.0%)	
Total (n)	10	141	

* Fisher exact test.

The overall *JAMA* scores of 151 websites were 2.2 ± 1.19 (median = 2, min = 0, max = 4). Of these websites, 57 (37.7%) were classified as high-reliable (*JAMA* score ≥ 3), whereas 94 (62.3%) were classified as low-reliable websites (*JAMA* score ≤ 2). The inter-rater reliability for determining the *JAMA* score was almost excellent (Cohen's kappa coefficient (κ) = 0.930). Among all the websites evaluated, 19 (12.6%) had HONcode stamp, whereas 132 (87.4%) were not certified by the stamp. When the typologies of websites were evaluated in terms of *JAMA* scores, 93.3% of scientific publishing websites, 59.1% of news websites, 59.0% of health portals, 22.2% of nonprofit websites, 17.9% of commercial websites, 16.7% of professional websites, 14.3% of government websites, and 0.0% of other websites were classified as high-reliable websites. Table 2 presents the *JAMA* scores and classifications according to the website typologies. When the *JAMA* scores of the first ten and remaining websites were compared, there was no significant difference between the groups (p = 0.623). Table 3 presents the comparison of *JAMA* scores of the initial ten and remaining websites.

**Table 2:** *JAMA* scores according to website typologies

Type	*JAMA* score Mean ± SD Median (Min-Max)	JAMA High-reliable n (%)	JAMA Low-reliable n (%)	p
Commercial	1.56 ± 0.94 1 (0-4)	7 (17.9%)	32 (82.1%)	<0.001*
Government	2.14 ± 0.9 2 (1-4)	1 (14.3%)	6 (85.7%)	
Health portal	2.95 ± 1.0 3 (1-4)	13 (59.0%)	9 (41.0%)	
News	2.64 ± 1.09 3 (0-4)	13 (59.1%)	9 (40.9%)	
Nonprofit	1.67 ± 1.04 2 (0-4)	6 (22.2%)	21 (77.8%)	
Professionals	1.78 ± 0.88 2 (1-4)	3 (16.7%)	15 (83.3%)	
Scientific publishing	3.73 ± 0.8 4 (1-4)	14 (93.3%)	1 (6.7%)	
Others	N/A	0 (0.0%)	1 (100.0%)	

* Fisher exact test (comparison of high-reliable and low-reliable websites).

*JAMA: Journal of the American Medical Association*; N/A: not applicable.

**Table 3:** *JAMA* scores of first ten and remaining

Websites	Initial 10 (n = 10) Mean ± SD Median (Min-Max)	Others (n = 141) Mean ± SD Median (Min-Max)	p
*JAMA* score	2.4 ± 1.26 2 (1-4)	2.19 ± 1.19 2 (0-4)	0.623#

# Mann-Whitney U test.

1 *JAMA: Journal of the American Medical Association.*

The mean FKG score was 8.81 ± 2.21 (median = 8.7, min = 2.2, and max = 16.9), and the mean SMOG score was 7.63 ± 1.81 (median = 7.4, min = 1.8, max = 14.1) in the evaluated websites. When the initial ten and remaining websites were compared in terms of readability scores, there was no difference for FKG and SMOG scores (p = 0.425 and 0.461, respectively). When websites were compared according to reliability in terms of readability, high-reliable websites had higher FKG and SMOG scores than low-reliable websites (p = 0.004 and < 0.001, respectively). Table 4 presents the between-group comparisons of readability scores. Additionally, we observed weak positive correlations between the *JAMA* scores and readability scores (r = 0.202, p = 0.013 for FKG and r = 0.283, p = <0.001 for SMOG).

**Table 4:** Comparison of readability scores of websites

	Mean ± SD Median (Min-Max)	Mean ± SD Median (Min–Max)	p
Websites on the first page and others	Initial 10 (n = 10)	Remaining websites (n = 141)	
FKG	9.12 ± 1.53 9 (7.1-11.3)	8.79 ± 2.25 8.5 (2.2-16.9)	0.425#
SMOG	7.74 ± 0.94 7.85 (6.3-9.3)	7.62 ± 1.86 7.4 (1.8-14.1)	0.461#
Websites according to *JAMA* score	High-reliable (n = 57)	Low-reliable (n = 94)	
FKG	9.47 ± 2.41 9.3 (4.8-16.8)	8.42 ± 1.99 8.1 (2.2-16.9)	0.004#
SMOG	8.23 ± 2.06 8 (1.8-14.1)	7.26 ± 1.53 7 (3.4-13.8)	<0.001#

# Mann-Whitney U test.

FKG: Flesch-Kincaid Grade; SMOG: Simple Measure of Gobbledygook score; *JAMA: Journal of the American Medical Association.*

## DISCUSSION

Internet is widely used by patients as a source of information (26). The ability to obtain medical information accurately, conveniently, and quickly helps patients to be better informed on decision making and participating. Tools such as search engines assist patients in finding related information online. However, finding relevant information may be challenging; also, the content of most websites may contain inconsistencies and inaccuracies. In this study, we aimed to evaluate whether online sources provide high-reliable and readable information about osteoporosis. We also sought to determine which types of sources provide more high-reliable information. Moreover, we attempted to ascertain whether there is a difference between websites on the first page and remaining pages in terms of reliability and readability. Nevertheless, we sought whether there is a correlation between the reliability and readability of the websites. A large number of websites were commercial, nonprofit websites, news, and health portals in our whole search process, whereas there were news, government, and nonprofit websites on the first page of Google. There was no difference between the initial ten websites and the remaining websites in terms of typology. Scientific publications, health portals, and news had high-reliable content, and in terms of *JAMA* scores, there was no difference between the first ten websites and the remaining websites. The FKG and SMOG readability scores were similar in the first ten pages and remaining pages. High-reliable pages had higher readability scores than low-reliable pages; hence, they were harder to read. There was a weak positive correlation between *JAMA* and readability scores.

There are many types of websites providing health-related information. We sorted these webpages into eight categories; however, even in the same category, there is a variety of styles for providing the information. Two independent investigators evaluated the website typology and inter-rater reliability for determining the typology, which was almost excellent (Cohen's κ = 0.954). The third investigator finalized the decision. Most of the health portals and news sites were administered by professional media corporations, which are aware of health-related writing rules. This practice resulted in higher *JAMA* scores for these typologies. Interestingly, government-supported websites and websites by professional organizations and individuals lacked high-reliable information. These websites mostly did not provide the authors’ name(s) or references by self-confidence. Even though readers may have to trust the information provided on these types of websites, correct referencing, dates, and author names need to be proven and provided. These websites should provide the readers with adequate information; thus, nonexpert readers may assess the reliability of information easily. There are concerns about websites with financial purposes for providing biased and incomplete or incorrect information (16). As legitimating these concerns, commercial websites mostly (82.1%) provided low-reliable information. Gladly, there was only one commercially purposed website in the initial ten websites. Many studies demonstrated that high rates of online information are related to commercial purposes (16,27,28). However, these rates were lower than those presented in our study. This result may be related to a different evaluation method. We accepted hospital webpages, which are offering an appointment with a physician or providing a telephone number for reservation after the information as webpages with a commercial purpose, which may be the reason for higher commercial rates.

Of the evaluated 151 websites, 57 (37.7%) were classified as high reliable, whereas 94 (62.3%) were classified as low reliable. The high rates of low *JAMA* scores represent that there is unreliable information on osteoporosis on the internet. Qualification, according to *JAMA* scores, vary in studies investigating online information on different topics. Arif and Ghezzi (14) reported a 37% rate of high-reliable websites on breast cancer-related information. In more recent studies, Basavakumar and cols. (13) reported a 43% rate of high-reliable websites on fibromyalgia, and Kocyigit and cols. (16) reported 46% rate of high-reliable websites on Ankylosing spondylitis. The presence of the HONcode stamp varied from 7% to 53% in the aforementioned studies. HONcode certification rate was 12.6% in our study, thereby indicating a low certification process. None of the scientific published materials had a HONcode stamp, which may be the cause of low rates. The necessity of a HONcode certification for scientific publishing is a matter of debate; however, adequate certification methods could be necessary for appropriate and truthful online information. A protocol or a committee to assess health information-related websites before they are accessible to the public may be considered.

Joshi and cols. (29) evaluated the nutrition-related information on osteoporosis in different websites. They entered five search terms in various search engines and categorized the websites according to their extensions (such as .gov, .edu, .com). They assessed the websites with a scale by the Agency for Health Care Policy and Research and found that .org and .com sites present better reliability of information about nutrition on osteoporosis than other websites. We disagree with this categorization. Recently, many nonprofit organizations use a website with .com extension, many websites with .com extension may provide better reliability of information than websites with .org extension. Another study by Mack and cols. (30) assessed the reliability and accuracy of online physical activity resources for osteoporosis. They also used multiple keywords for their search and demonstrated a low accuracy of online information. In their study, just over half of the websites indicated high-reliable information. They concluded that accurate physical activity information on the web might contribute to better health and well-being of patients with osteoporosis.

Wallace and cols. (9) evaluated the readability and reliability of web-based osteoporosis information with suitability assessment of materials (SAM) and DISCERN tools. They searched the first 30 websites indexed in three popular search engines of that time and evaluated 27 websites. They determined that 51.9% of online materials were not suitable according to SAM and DISCERN tools for treatment options, as these contents indicated inadequate reliability. Also, they found out that 82.6% of the materials were unavailable for reading with an average level at a grade of 11.5 ± 2.8. They concluded that web-based information is above the reading ability of most American adults, and much of this information has inadequate reliability. We found out that there were 57 (37.7%) high-reliable websites among the evaluated websites. Regarding the increase of online material since 2005, we expected a decrease in high-reliable online material. In our study, FKG scores [8.81 ± 2.21 (median = 8.7, min = 2.2, max = 16.9)] were below the ninth grade, which is considered to be easily understood by 13- to 15-year-old students (31). The mean SMOG scores were 7.63, which indicates that seven to eight years of education is necessary for reading the texts. On the contrary, the FKG score [9.47 ± 2.41 (median = 9.3, min = 4.8, and max = 16.8)] and the SMOG score [8.23 ± 2.06 (median = 8, min = 1.8, max = 14.1)] were increased when we assessed the high-reliable websites, thereby indicating a statistical difference between high- and low-reliable websites (p = 0.004 for FKG and p < 0.001 for SMOG). This observation demonstrates that the high-reliable websites are challenging to read, and the owners of the websites may pay attention to ease the readability in their online materials. We also sought to determine a correlation between reliability scores and readability of online texts. As a result, we found a weak positive correlation; however, it is not possible to fully conclude that all of the high-reliable materials are hard to read.

Our study is not without limitations. Firstly, we only searched for one word, “osteoporosis”. The results may be different with more keywords. There are various methodological evaluations of online information. We used one of the methodologies employed in recent studies (13,14,16), but results might be different with different methods. The *JAMA* scoring system, used in this study, also has some limitations. It does not directly assess the content accuracy of online information, and some websites which have rich content and high-quality information may present lower scores in the evaluation. In this study, we did not evaluate the accuracy of the content, which may be subject to another study. Moreover, we only evaluated websites that were English. Finally, the geographic location, where website searches were performed, might have affected the results.

In conclusion, most of the scientific published materials, news, and health portals provide high-reliable information. Among 200 websites, the initial (first) ten have similar scores with the remaining websites in terms of reliability and readability. Most of the materials are below the reading ability of an average American adult. High-reliable material is harder to read, and there is a weak correlation between *JAMA* scores and higher readability scores. The website providers should thoroughly consider reliability and readability and establish more broad-based certification methods for health-related information on the World Wide Web.
